# Tobacco growing and the sustainable development goals, Malawi

**DOI:** 10.2471/BLT.16.175596

**Published:** 2017-02-09

**Authors:** Margarete C Kulik, Stella Aguinaga Bialous, Spy Munthali, Wendy Max

**Affiliations:** a Center for Tobacco Control Research and Education, 530 Parnassus Avenue, Suite 366, University of California San Francisco, San Francisco, CA 94143-1390, United States of America (USA).; b School of Nursing, University of California San Francisco, San Francisco, USA.; c Chancellor College, University of Malawi, Zomba, Malawi.

## Abstract

Negative impacts of tobacco result from human consumption and from tobacco-growing activities, most of which now occur in low- and middle-income countries. Malawi is the world’s largest producer of burley tobacco and its population is affected by the negative consequences of both tobacco consumption and production. In countries like Malawi, tobacco control refers to control of the tobacco supply chain, rather than control of consumption. We review the impact of tobacco cultivation, using Malawi as an example, to illustrate the economic, environmental, health and social issues faced by low- and middle-income countries that still produce significant tobacco crops. We place these issues in the context of the sustainable development goals (SDGs), particularly 3a which calls on all governments to strengthen the implementation of the World Health Organization Framework Convention on Tobacco Control. Other goals address the negative effects that tobacco cultivation has on development. The SDGs offer an opportunity for low- and middle-income countries that are dependent on tobacco production and that are not yet parties to the Convention, to reconsider joining the FCTC.

## Résumé

Les impacts négatifs du tabac résultent de la consommation humaine et des activités de culture du tabac, qui sont aujourd'hui exercées pour la plupart dans les pays à revenu faible et intermédiaire. Le Malawi est le plus grand producteur mondial de tabac Burley et sa population subit les effets négatifs de la consommation et de la production de tabac. Dans des pays comme le Malawi, le contrôle du tabac porte davantage sur le contrôle de la chaîne d'approvisionnement que sur le contrôle de la consommation de tabac. Nous examinons ici l'impact de la culture du tabac, en prenant le Malawi comme exemple pour illustrer les problèmes économiques, environnementaux, sanitaires et sociaux que rencontrent les pays à revenu faible et intermédiaire qui continuent de produire d'importantes récoltes de tabac. Nous plaçons ces problèmes dans le cadre des objectifs de développement durable (ODD), en particulier du 3.a, qui appelle tous les gouvernements à renforcer l'application de la Convention-cadre de l'Organisation mondiale de la Santé pour la lutte antitabac (CCLAT). D'autres objectifs s'intéressent aux effets négatifs de la culture du tabac sur le développement. Les ODD offrent l'opportunité aux pays à revenu faible et intermédiaire qui dépendent de la production de tabac et qui ne sont pas encore parties à la Convention de réenvisager de la signer.

## Resumen

Los impactos negativos del tabaco se derivan del consumo humano y las actividades de cultivo de tabaco, la mayoría de las cuales suelen realizarse actualmente en países con ingresos bajos y medios. Malawi es el mayor productor de tabaco burley del mundo y su población se ha visto afectada por las consecuencias negativas del consumo y la producción de tabaco. En países como Malawi, el control del tabaco hace referencia al control de la cadena de suministro de tabaco, en lugar del control del consumo. Se revisó el impacto del cultivo de tabaco, utilizando Malawi como ejemplo, para ilustrar los problemas económicos, medioambientales, sanitarios y sociales a los que se enfrentan los países de ingresos bajos y medios que siguen produciendo grandes cosechas de tabaco. Se situaron estos problemas en el contexto de los objetivos de desarrollo sostenible, principalmente el 3.a, que hace un llamamiento a todos los gobiernos para que fortalezcan la implementación del Convenio Marco de la OMS para el Control del Tabaco (CMCT). Otros objetivos abordan los efectos negativos del cultivo de tabaco en el desarrollo. Los ODS ofrecen una oportunidad para que los países con ingresos bajos y medios que dependen de la producción del tabaco y que aún no forman parte del convenio reconsideren incorporarse al CMCT.

## ملخص

تنشأ الآثار السلبية للتبغ من الاستهلاك البشري وبفعل أنشطة زراعة التبغ والتي يقع أغلبها الآن في البلدان منخفضة ومتوسطة الدخل. وتعتبر ملاوي أكبر دول العالم إنتاجًا للتبغ من نوع بيرلي، ويتأثر سكانها بالآثار السلبية الناتجة عن استهلاك التبغ وإنتاجه. وفي بلدان كملاوي، تشير الرقابة على التبغ إلى الرقابة المفروضة على سلسلة الإمداد والتوزيع، بدلاً من الرقابة على الاستهلاك. وباستخدام دولة ملاوي كمثال قمنا بإجراء مراجعة لآثار زراعة التبغ وذلك لتوضيح المشكلات الاقتصادية والبيئية والصحية والاجتماعية التي تواجهها البلدان منخفضة ومتوسطة الدخل والتي ما تزال تقوم بإنتاج محاصيل التبغ بكميات كبيرة. وقد قمنا بوضع هذه المشكلات في إطار أهداف التنمية المستدامة وخاصة الهدف 3-أ والذي يدعو كل الحكومات إلى دعم تنفيذ الاتفاقية الإطارية لمنظمة الصحة العالمية والمعنية بمكافحة التبغ (FCTC). وقد ركزت الأهداف الأخرى على الآثار السلبية التي تمس التنمية بسبب زراعة التبغ. وتعمل أهداف التنمية المستدامة على تقديم فرصة للبلدان منخفضة ومتوسطة الدخل – التي تعتمد على انتاج التبغ ولم تصبح بعد طرفًا في الاتفاقية الإطارية لمكافحة التبغ – لإعادة النظر في الانضمام إليها.

## 摘要

烟草的负面影响是由人类对烟草的消费及种植活动造成的。如今,大多数负面影响发生在中低收入国家。 马拉维是世界上最大的白肋烟生产商,其人民就受到了烟草消费及生产的负面影响。 在马拉维等国家,烟草控制指对烟草供应链进行控制,而非控制消费。 我们以马拉维为实例,评审了其种植烟草的影响,以阐明仍在大量种植烟草作物的中低收入国家所面临的经济、环境、健康及社会问题。 我们将这些问题置于可持续发展目标的背景下,特别是呼吁所有政府加强实施世界卫生组织《烟草控制框架公约》(FCTC) 的 3.a 目标上。 其它目标旨在解决烟草种植对发展造成的负面影响。 联合国可持续发展目标 (SDG) 向依赖烟草生产且尚未加入公约的中低收入国家提供重新考虑加入《烟草控制框架公约》(FCTC) 的机会。

## Резюме

Негативное воздействие табака происходит в результате его употребления человеком, а также в результате деятельности в области табаководства, которая в настоящее время по большей части ведется в странах с низким и средним уровнем дохода. Малави является крупнейшим производителем в мире табака сорта Берли (Burley), и его население страдает от негативных последствий как потребления табака, так и его производства. В таких странах, как Малави, контроль над табаком сводится к контролю цепи поставок табачных изделий, а не к контролю потребления табака. Авторы рассматривают влияние выращивания табака на примере Малави, чтобы проиллюстрировать экономические, экологические, медицинские и социальные проблемы, с которыми сталкиваются страны с низким и средним уровнем дохода, которые до сих пор производят крупные урожаи табака. Эти проблемы помещаются в контекст целей в области устойчивого развития (ЦУР), особенно цели 3.а, в которой заложен призыв к правительствам всех стран активизировать осуществление Рамочной конвенции Всемирной организации здравоохранения по борьбе против табака (РКБТ). Другие цели связаны с устранением негативных последствий, которые оказывает выращивание табака. Цели в области устойчивого развития предлагают возможность для стран с низким и средним уровнем дохода, которые зависят от производства табака и которые еще не являются участниками этой конвенции, рассмотреть присоединение к РКБТ ВОЗ.

## Introduction

The World Health Organization (WHO) estimates that, worldwide, over 1 billion people smoke and over 6 million die each year from tobacco-related causes.^1^ Most tobacco users live in low- or middle-income countries.^2^ Tobacco production, which now mostly occurs in low- and middle-income countries,^3^ has negative effects that extend beyond those on health, to economic problems and environmental degradation.

Malawi, which is the world’s largest producer of burley leaf tobacco^4^^,^^5^ and also one of the poorest countries in the world,^6^ is particularly badly affected by the multiple negative consequences of tobacco consumption and production but has few policies on tobacco control.^7^ In Malawi, tobacco control does not primarily mean control of tobacco consumption – as in most high-income countries with high levels of tobacco use. Only 5% of the country’s tobacco crop is consumed domestically.^5^ It has been estimated that, in Malawi in 2013, about 18–22% of men but only 1–3% of women smoked daily.^8^^,^^9^ A study in the capital city of Lilongwe found that, while only 9.1% of males and 2.8% of females aged 13–15 years reported being current smokers in 2001–2002, 28.6% and 10.1%, respectively, had tried smoking.^10^ Concerns exist that smoking is becoming more common among Malawian adolescents as a result of increasing exposure, via the Internet and television, to tobacco advertising and tobacco use.^10^ In a study of Malawian college students in 2009, 29% of the male students and 18% of the female students reported that they were smokers.^11^ The country’s polices are mainly to control the tobacco supply chain. Malawi, as other low- or middle-income countries, struggles to resist tobacco industry influence and to develop sustainable alternatives to tobacco production.^12^^,^^13^

In this article, we use Malawi – a country that is not a party to the WHO Framework Convention on Tobacco Control (FCTC)^14^ – as a case study to illustrate the tensions between economic reliance on tobacco production and the negative impacts of such reliance on the economies of low- and middle-income countries. In reviewing the opportunities for improving tobacco control globally, we highlight the importance of addressing economic dependence on tobacco production – within the context of the sustainable development goals (SDGs).^15^

## Tobacco cultivation

### 

In 2013, the seven largest tobacco producers in the world were, in decreasing levels of production: China, Brazil, India, the United States of America, Indonesia, Zimbabwe and Malawi.^3^ Malawi produced about 133 000 tonnes of tobacco leaf in 2013 – down from a high of 208 000 tonnes in 2009 and up from a low – as recorded by the Food and Agriculture Organization of the United Nations – of 73 000 tonnes in 2012.^3^ While annual production increased to about 160 000 tonnes in 2014–2015, prices have been falling since 2013 (Malawi Tobacco Control Commission, unpublished data, 2015). There are multiple potential reasons for the decreasing prices: farmers may have been selling more tobacco than the quotas established by leaf-buying companies; some of the tobacco produced has been of poor quality; more tobacco on the international markets has come from competing countries; there has been a decrease in global demand as the result of international efforts in tobacco control; and tobacco companies – which use inventory strategies that encourage the release of stocks when it suits the companies’ economic needs – have released stock. In Malawi, as the prices of tobacco leaf have fallen, the costs of tobacco production have gone up because the government has stopped subsidizing the costs of fertilizer. Such volatility in tobacco production is commonly observed.^16^

The tobacco industry in Malawi is dominated by two global leaf-buying companies: Alliance One International and Universal Corporation – represented nationally as Alliance One Malawi and Limbe Leaf, respectively. These two companies sell tobacco leaf to British American Tobacco, Imperial Tobacco, Japan Tobacco and Philip Morris International.^17^ The ways in which the tactics of such global actors in the tobacco industry influence policy-making in Malawi have been described elsewhere.^17^^–^^21^ There are also allegations of a close relationship between the industry and many government officials who own – or have other financial interests in – estates on which tobacco is grown.^5^

As tobacco is the major source of foreign exchange in Malawi,^22^ shortfalls in the national tobacco crop and/or low prices for tobacco in world markets have an immediate negative effect on the national economy. The low sales volume in 2012 resulted in a shortfall in the cash available for imports such as fuel.^23^ Although the tobacco companies in Malawi have been accused of colluding to maintain artificially low tobacco prices,^19^ the companies have blamed external forces for the depressed prices. Malawi’s president between 2004 and 2012, Bingu wa Mutharika, repeatedly attempted to impose price floors on Malawi tobacco, but was thwarted when the transnational tobacco companies refused to buy tobacco leaf from Malawi unless such floors were removed.^24^

### Tobacco farmers

The best available estimates suggest that about 60% of Malawi’s burley tobacco farmers are smallholders who own their land and 30% are estate or tenant farmers who work on land that they do not own.^17^ The tenant farmers who work on large tobacco estates are often trapped in a cycle of poverty. Each year, they are usually lent the money to pay for crop inputs and maize to feed their families; once their crop has been harvested and sold they are paid whatever is left after subtracting the loan. As the sale of the tobacco is often insufficient to cover the loan, many tenants start the next season in debt.^25^^,^^26^ This cycle makes it difficult for tenant farmers to ever improve their economic circumstances. The vulnerability of tobacco tenants is reflected in their low educational attainment. In a study of such tenants in Malawi in 2000, more than one quarter had no education, 40% reported they could not read, 40% reported they could not write and over 40% said they could not work with numbers.^25^ Since 2005, contract farming – in which farmers sell directly to leaf buyers who also offer credit and technical support – has become more common in Malawi.^27^ As farmers who enter into contracts with industry tend to become indebted quickly, it is worrying that, by 2010, an estimated 10% of the burley tobacco farmers in Malawi had become contract farmers.^17^^,^^26^

The tobacco production process has negative health impacts on tobacco workers. Each day, a tobacco worker who plants, cultivates and harvests tobacco may absorb as much nicotine as found in 50 cigarettes.^28^ In consequence, tobacco workers may suffer from the nicotine poisoning known as green tobacco sickness.^28^ Given the common physical requirements of tobacco production, it also seems likely that tobacco workers in Malawi suffer similar musculoskeletal injuries to those recorded among tobacco workers in the United States.^29^^,^^30^

Tobacco farming is particularly devastating for the farmers’ children. They work in the fields to help their families increase production and are at risk from green tobacco sickness, being injured and/or missing days of school because of illness or farming needs. Children are particularly vulnerable to green tobacco sickness because of their size relative to the amount of nicotine absorbed.^28^ Their poor schooling makes it impossible for them to break out of the cycle of poverty. Although Malawi’s Employment Act of 2000 prohibits children younger than 14 years from working, it is not enforced.^21^ It has been estimated that 80 000 children work in tobacco farming in Malawi.^31^ Poor education is common among children in rural Malawi: although 90% of rural children from all wealth quintiles attend primary school, only 8.8% attend secondary school and the corresponding rates for the lowest wealth quintile are even lower, at 83.2% and 3.2%, respectively.^32^ Child labour itself is destructive for those concerned, and it has been estimated that the tobacco industry in Malawi annually saved the equivalent of 10.7 million United States dollars (US$) through unpaid child labour from 2000–2010.^18^

### Environmental effects

Tobacco cultivation also leads to large-scale environmental degradation. Even though most of Malawi’s tobacco is air-cured, the annual cost of tobacco-related deforestation in Malawi has been estimated at US$ 6.4 million – based on the hectares of land cleared for tobacco production.^18^ Such costs are incurred despite the tobacco industry’s reforestation programmes – which often involve the planting of non-native species to be used in the flue-curing of tobacco.^18^ The pesticide use that accompanies tobacco production causes environmental pollution that reduces animal and plant biodiversity and damages farmers’ health.^33^^,^^34^ The tobacco industry has recommended that Kenyan tobacco farmers make as many as 16 applications of pesticide during the first few months of seedling production.^35^

### The FCTC and the SDGs

The FCTC, which is the first international public health treaty, focuses on curbing the demand and supply of tobacco.^14^ It provides technical guidance in all aspects of tobacco control. Articles 17 and 18 focus, respectively, on supporting alternative, sustainable livelihoods for farmers growing tobacco and protecting the environment and the health of those involved in tobacco production.^36^^,^^37^ Among the tobacco-growing countries in Africa, Malawi and Mozambique are the only two that are not parties to the FCTC. Zimbabwe became a party in December 2014. A growing body of evidence is linking the implementation of the FCTC with improved sustainable development^38^ – a relevant issue as the world embraces the United Nations’ 2030 agenda for sustainable development.^39^ This agenda, which is a global framework, builds upon the millennium development goals (MDGs),^40^ that were in place between 2000 and 2015 and mostly focused on human development and poverty reduction in low- and middle-income countries. The SDGs, which form the core of the agenda, include a set of 17 aims and 169 specific targets.^15^ The SDGs are broader than the MDGs and are focused more on global sustainable development. The main implicit aim of the SDGs is to improve the general life expectancies, education and incomes of people living in resource-poor countries, as reflected in an improved human development index (HDI) score.^41^

The SDGs advocate for safe and sustainable production; promote environmental responsibility; and strive to eliminate hunger and reduce inequalities. They offer a unique opportunity to address the harms of tobacco throughout the supply chain.^42^ SDG 3, which advocates for healthy lives, is particularly important in this respect. Specific target 3.4 aims to reduce premature mortality from noncommunicable diseases by one third by 2030.^15^ Target 3a advocates for the strengthening of the implementation of the FCTC in all countries as appropriate.^15^ We now have opportunities both for the FCTC to act as a catalyst for sustainable development and for interventions, policies and strategies based on the SDGs to support tobacco control in those resource-poor countries that currently lack tobacco-control capacities.

Countries that are currently not parties to the FCTC are unlikely to benefit from global efforts to implement FCTC Articles 17 and 18 and might face growing tobacco-related economic, environmental and social problems. As a party to the FCTC, a country may obtain external technical assistance for its tobacco control efforts^14^ as well as financial assistance, when – and if – it becomes available. Malawi and Mozambique already have a very poor HDI – in the 2014 HDI listing that covered 188 countries, they were ranked 173 and 180, respectively^41^ – and high proportions of their populations are undernourished. Several other African countries are major tobacco growers; for instance, Uganda and Zambia combined produced 39% of all global burley leaf exports in 2011,^17^ and in 2012 the United Republic of Tanzania and Zimbabwe were also among the top 25 countries producing raw tobacco.^3^ But, in terms of its overall economy, Malawi depends on tobacco as a cash crop more than all of them.^8^^,^^16^

## Discussion

The specific issues discussed here are not limited to Malawi but are also applicable to other tobacco-growing low- and middle-income countries where the far-reaching negative impacts of tobacco production are particularly troublesome.^43^ All such countries could benefit greatly from the successful integration of tobacco control in the implementation of the SDGs. The SDGs give a prominent position to the FCTC, within health-related SDG 3, and many of the remaining 16 goals are directly related to the negative effects of tobacco cultivation on development. Overall, work towards the SDGs should help reduce a country’s economic dependence on tobacco, reduce tobacco’s negative health impacts, reduce the number of families trapped in a cycle of poverty, improve food security and reduce environmental degradation. Through support for alternative, sustainable livelihoods for tobacco growers – i.e. Article 17 of the FCTC – a country could help meet three SDGs: SDG 2, which advocates for the end of hunger and the promotion of food security, nutrition and sustainable agriculture; SDG 12, which encourages sustainable consumption and production patterns; and SDG 15, which advocates against deforestation and land degradation. Some successful examples exist of implementing programmes to explore a switch from tobacco to other crops. A programme in Brazil encourages a shift from tobacco farming to fruit or vegetable cultivation. The new fruit and vegetable growers are guaranteed a market for their produce via a government programme that promotes the purchase of locally-grown fruit and vegetables for school lunches.^44^ The specific problems that lead families to remain trapped in a cycle of poverty are echoed in SDG 4, which is aimed at education and lifelong learning opportunities, and SDG 8, which promotes economic growth through productive employment and decent work for all. In striving to attain all of these goals, a country’s economic dependence on growing tobacco could be reduced.^42^ A so-called road map, released at the FCTC’s Conference of the Parties in October 2014, provides alternatives to tobacco growing and ways to protect the environment and the health of tobacco workers.^36^^,^^37^

Without external assistance, Malawi has relatively limited capacity to develop alternatives to tobacco production that are economically viable. The country could benefit greatly from becoming a party to the FCTC and could then have the opportunity to receive assistance through the incorporation of the FCTC into the SDGs. A major shortcoming of the MDGs, which impaired more effective international tobacco control, was their omission of noncommunicable diseases and tobacco control.^45^ Because of this omission, assistance funding for tobacco control has been difficult to obtain through international aid mechanisms focused on development. In addition, the funding system for tobacco control has been skewed towards demand issues rather than supply issues. 

Another problem, at least for tobacco-growing countries in Africa, is that many believe the current methods of disbursing aid to Africa are suboptimal^46^ and that, in general, there needs to be a move towards more independence from donor assistance. National capacity could be increased by fostering cooperation among officials in the ministries and civil society groups that aim to protect public health and by promoting alternative livelihoods for tobacco farmers. A multisectoral approach that tackles all the complexities of economic development is needed. Even though the path to full international FCTC implementation remains long, low- and middle-income countries that are not parties might be left even further behind. While being a party to the FCTC does not guarantee funding for tobacco control, it should facilitate access to such funding – and to technical assistance and so-called south–south collaboration. Successful implementation of the FCTC’s recommended taxation of tobacco products might help to pay for a country’s tobacco control efforts. It would also generate funds for the implementation of the SDGs overall – in a use of tobacco taxes that has been promoted by WHO.^47^

Some encouraging evidence exists that the governmental narrative in Malawi is changing. The Malawi development report for 2006–2011^48^ stated that “Malawi will maintain a position of market leader in burley tobacco … Malawi will also increase production … [and will be] exploring additional markets for tobacco, including tobacco products ….” However, the corresponding report for 2011–2016^49^ has a different tone: “… the objective will be to increase the country’s market share in traditional agricultural products such as sugar, cotton, coffee and tea as well as diversifying away from tobacco into wheat, cassava, macadamia nuts, fruits, pulses and vegetable commodities among others. … [as] On the export market, the sub-sector is currently dominated by tobacco which is facing problems due to the anti-smoking lobby. Within agriculture, it is therefore important for the country to diversify its export base away from tobacco.”

Perhaps in contrast to this encouraging trend, an annual report for 2014 revealed that the tobacco industry, via the participation of Alliance One Malawi, was directly involved in the G8 New Alliance for Food Security and Nutrition^50^ – a public–private partnership of the Government of Malawi, the European Union, several development agencies and companies from the private sector.

If followed, the approach suggested in the Malawi development report for 2011–2016 should convey benefits for Malawi – by reducing economic dependence on tobacco, broadening the country’s agricultural and economic base and reducing the health risks associated with both the consumption and the production of tobacco. A next step could be to accede to the FCTC and increase the benefits from the sustainable development agenda.

## Conclusion

A multipronged approach to tobacco control in the low-and middle-income countries of sub-Saharan Africa, that incorporates both tobacco consumption and production, is needed. Although the increasing prevalence of smoking – and the associated future health issues – cannot be ignored, all the problems related to tobacco production must also be tackled. Although being a party to the FCTC might not be a sufficient motivator for the successful implementation of tobacco control efforts in low- and middle-income countries, the FCTC offers incentives to parties that implement its policies. The SDGs stress the importance of the implementation of those policies, address many of the economic, environmental, health and social issues caused by tobacco cultivation and offer a new opportunity for low- and middle-income countries that are dependent on tobacco production but not currently parties to the FCTC to reconsider joining the treaty.

## References

[1] WHO report on the global tobacco epidemic, 2013: enforcing bans on tobacco advertising, promotion and sponsorship. Geneva: World Health Organization; 2013. Available from: http://apps.who.int/iris/bitstream/10665/85380/1/9789241505871_eng.pdf?ua=1 [cited 2017 Jan 23].

[2] WHO report on the global tobacco epidemic, 2015: raising taxes on tobacco. Geneva: World Health Organization; 2015. Available from: http://apps.who.int/iris/bitstream/10665/178574/1/9789240694606_eng.pdf?ua=1&ua=1 [cited 2017 Jan 23].

[3] FAOSTAT [Internet]. Rome: Food and Agriculture Organization of the United Nations. Available from: http://www.fao.org/faostat/en/#data/countries_by_commodity/visualize [cited 2015 Mar 15].

[4] Bickers C. Is the leaf pendulum swinging away from undersupply? Tobacco International. New York: Lockwood Publications; 2008. Available from: http://www.tobaccointernational.com/1208/feature2.htm[cited 2015 Mar 15].

[5] Makoka D, Munthali K, Drope J. Malawi. In: Drope J, editor. Tobacco control in Africa: people, politics and policies. Ottowa: International Development Research Centre; 2011. pp. 167–83. Available from: http://idl-bnc.idrc.ca/dspace/bitstream/10625/47373/1/IDL-47373.pdf[cited 2015 Jan 15].

[6] GDP per capita (current US$) [Internet]. Washington: World Bank; 2014. Available from: http://data.worldbank.org/indicator/NY.GDP.PCAP.CD?end=2014&locations=MW&order=wbapi_data_value_2014+wbapi_data_value+wbapi_data_value-last&sort=desc&start=2010 [cited 2015 Jul 15].

[7] WHO report on the global tobacco epidemic, 2015: country profile Malawi. Geneva: World Health Organization; 2015. Available from: http://www.who.int/tobacco/surveillance/policy/country_profile/mwi.pdf?ua=1, [cited 2016 Dec 15].

[8] Eriksen M, Mackay J, Schluger N, Gomeshtapeh FI, Drope J. The tobacco atlas. 5th ed. Atlanta: American Cancer Society; 2015.

[9] Status of tobacco production and trade in Africa - factsheets. Geneva: World Health Organization; 2015. Available from: http://apps.who.int/iris/bitstream/10665/203234/1/9789241565127_eng.pdf?ua=1 [cited 2016 Apr 15].

[10] Muula AS, Mpabulungi L. Cigarette smoking prevalence among school-going adolescents in two African capital cities: Kampala Uganda and Lilongwe Malawi. Afr Health Sci. 2007 3;7(1):45–9. 17604526 10.5555/afhs.2007.7.1.45 PMC2366124

[11] Kasapila W, Mkandawire TS. Drinking and smoking habits among college students in Malawi. Eur J Soc Sci. 2010;15(3):441–8.

[12] Jakpor P. Nigeria: how British American Tobacco undermines the WHO FCTC through agricultural initiatives: invited commentary. Tob Control. 2012 3;21(2):220. 10.1136/tobaccocontrol-2011-050411 22345252

[13] Patel P, Collin J, Gilmore AB. “The law was actually drafted by us but the Government is to be congratulated on its wise actions”: British American Tobacco and public policy in Kenya. Tob Control. 2007 2;16(1):e1. 10.1136/tc.2006.016071 17297056 PMC2598451

[14] WHO Framework Convention on Tobacco Control [Internet]. Geneva: World Health Organization; 2015. Available from: http://www.who.int/fctc/en/ [cited 2015 Apr 15].

[15] Open Working Group proposal for sustainable development goals. New York: United Nations; 2014. Available from: https://sustainabledevelopment.un.org/content/documents/1579SDGs%20Proposal.pdf [cited 2015 Apr 15].

[16] Observatory of economic complexity [Internet]. Cambridge: MIT Media Lab; 2015. Available from: http://atlas.media.mit.edu/en/visualize/tree_map/hs92/export/mwi/all/show/2012/ [cited 2015 Mar 15].

[17] Otañez M, Graen L. ‘Gentlemen, why not suppress the prices’: global leaf companies harm rural livelihoods in Malawi. In: Lappan W, Lecours N, Buckles D, editors. Tobacco control and tobacco farming: separating myth from reality. Ottawa: International Development Research Centre; 2014. pp. 61–95. Available from: http://idl-bnc.idrc.ca/dspace/bitstream/10625/53191/1/IDL-53191.pdf[cited 2015 Mar 15].

[18] Otañez M, Glantz SA. Social responsibility in tobacco production? Tobacco companies’ use of green supply chains to obscure the real costs of tobacco farming. Tob Control. 2011 11;20(6):403–11. 10.1136/tc.2010.039537 21504915 PMC3155738

[19] Otañez MG, Mamudu H, Glantz SA. Global leaf companies control the tobacco market in Malawi. Tob Control. 2007 8;16(4):261–9. 10.1136/tc.2006.019273 17652242 PMC2598545

[20] Otañez MG, Mamudu HM, Glantz SA. Tobacco companies’ use of developing countries’ economic reliance on tobacco to lobby against global tobacco control: the case of Malawi. Am J Public Health. 2009 10;99(10):1759–71. 10.2105/AJPH.2008.146217 19696392 PMC2741530

[21] Otañez MG, Muggli ME, Hurt RD, Glantz SA. Eliminating child labour in Malawi: a British American Tobacco corporate responsibility project to sidestep tobacco labour exploitation. Tob Control. 2006 6;15(3):224–30. 10.1136/tc.2005.014993 16728754 PMC2564665

[22] Davies P. Malawi: addicted to the leaf. Tob Control. 2003 3;12(1):91–3. 10.1136/tc.12.1.91 12612371 PMC1759074

[23] Malawi tobacco sales register success amid challenges - TCC. Nyasa Times. 5 April, 2013. Available from: http://www.nyasatimes.com/malawi-tobacco-sales-register-success-amid-challenges-tcc/ [cited 2017 Jan 23].

[24] Chapulapula T, Daniels G, Sole S. Tobacco helps ignite mass anger. Mail & Guardian. 1 August, 2011. Available from: http://mg.co.za/article/2011-07-29-tobacco-helps-ignite-mass-anger [cited 2017 Jan 23].

[25] Torres L. The smoking business. Tobacco workers in Malawi. Oslo: Fafo Institute for Applied Social Science; 2000.

[26] Tobacco production and tenancy labour in Malawi - treating individuals and families as mere instruments of production. Lilongwe: Centre for Social Concern; 2015.

[27] Makoka D, Drope J, Appau A, Labonte R, Li Q, Goma F, et al. Costs, revenues and profits: an economic analysis of smallholder tobacco farmer livelihoods in Malawi. Tob Control. 2016 10 8. 10.1136/tobaccocontrol-2016-053022 PMC5957074 29066593

[28] McKnight RH, Spiller HA. Green tobacco sickness in children and adolescents. Public Health Rep. 2005 Nov-Dec;120(6):602–5. 16350329 10.1177/003335490512000607 PMC1497768

[29] Pugh KJ, Pienkowski D, Gorczyca JT. Musculoskeletal trauma in tobacco farming. Orthopedics. 2000 2;23(2):141–3. 10688290 10.3928/0147-7447-20000201-14

[30] Lecours N. The harsh realities of tobacco farming: a review of socioeconomic, health and environmental impacts. In: Lappan W, Lecours N, Buckles D, editors. Tobacco control and tobacco farming: separating myth from reality. Ottawa: International Development Research Centre; 2014. p. 99–137. Available from: http://idl-bnc.idrc.ca/dspace/bitstream/10625/53191/1/IDL-53191.pdf [cited 2015 Mar 15].

[31] Palitza K. Child labour: the tobacco industry's smoking gun. The Guardian. 2011 Sep 14. Available from: https://www.theguardian.com/global-development/2011/sep/14/malawi-child-labour-tobacco-industry [cited 2015 Mar 15].

[32] Malawi demographic and health survey 2010. Calverton: ICF Macro; 2011.

[33] Kagaruki LK. Community-based advocacy opportunities for tobacco control: experience from Tanzania. Glob Health Promot Educ. 2010 6;17(2) Suppl:41–4. 10.1177/1757975910363932 20595340

[34] Lecours N, Almeida GE, Abdallah JM, Novotny TE. Environmental health impacts of tobacco farming: a review of the literature. Tob Control. 2012 3;21(2):191–6. 10.1136/tobaccocontrol-2011-050318 22345244

[35] Madeley J. Big business, poor peoples: the impact of transnational corporations on the world's poor. London: Zed Books; 1999.

[36] Economically sustainable alternatives to tobacco growing (in relation to Articles 17 and 18 of the WHO Framework Convention on Tobacco Control). Report by the working group. Geneva: World Health Organization; 2014. Available from: http://apps.who.int/gb/fctc/PDF/cop6/FCTC_COP6_12-en.pdf [cited 2015 Mar 15].

[37] Second report of Committee A. (Draft). Geneva: World Health Organization; 2016. Available from: http://www.who.int/fctc/cop/cop7/FCTC_COP_COMMITTEE_A_2.pdf [cited 2016 Dec 15].

[38] Development planning and tobacco control - Integrating the WHO Framework Convention on Tobacco Control into UN and national development planning instruments. New York: United Nations Development Programme; 2014.

[39] Resolution A/RES/70/1. Transforming our world: the 2030 agenda for sustainable development. In: Seventieth United Nations General Assembly, New York, 25 September 2015. New York: United Nations; 2015. Available from: http://www.un.org/ga/search/view_doc.asp?symbol=A/RES/70/1&Lang=E [cited 2017 Feb 3].

[40] Millennium development goals. New York: United Nations; 2014. Available from: http://www.un.org/millenniumgoals/ [cited 2015 Apr 15].

[41] Human development reports. Table 1: human development index and its components. New York: United Nations Development Programme; 2017. Available from: http://hdr.undp.org/en/composite/HDI [cited 2017 Jan 23].

[42] von Eichborn S, Abshagen M-L. Tobacco: antisocial, unfair, harmful to the environment. Tobacco production and consumption as an example of the complexity of sustainable development goals (SDGs). Berlin: Bread for the World - Protestant Development Service; 2015. Available from: https://www.unfairtobacco.org/wp-content/uploads/tobacco_antisocial_web.pdf [cited 2015 Jul 15].

[43] Hu TW, Lee AH. Tobacco control and tobacco farming in African countries. J Public Health Policy. 2015 2;36(1):41–51. 10.1057/jphp.2014.47 25428192 PMC4412848

[44] Eidt Goncalves de Almeida G. Diversification strategies for tobacco farmers: lessons from Brazil. In: Lappan W, Lecours N, Buckles D, editors. Tobacco control and tobacco farming: separating myth from reality. Ottawa: International Development Research Centre; 2014. pp. 211–45. Available from: http://idl-bnc.idrc.ca/dspace/bitstream/10625/53191/1/IDL-53191.pdf[cited 2015 Mar 15].

[45] Collin J. Tobacco control, global health policy and development: towards policy coherence in global governance. Tob Control. 2012 3;21(2):274–80. 10.1136/tobaccocontrol-2011-050418 22345267 PMC4474152

[46] Easterly WR, editor. Reinventing foreign aid. 1st ed. Cambridge: MIT Press; 2008.

[47] Tobacco taxes can be used to finance the sustainable development goals. The economic and health benefits of tobacco taxation. Geneva: World Health Organization; 2015. Available from: http://www.who.int/fctc/mediacentre/news/2015/sdg2015/en/ [cited 2015 Jun 15].

[48] Malawi growth and development strategy. From poverty to prosperity. 2006-2011. Lilongwe: Government of Malawi; 2006.

[49] Malawi growth and development strategy II 2011-2016. Lilongwe: Ministry of Agriculture and Irrigation; 2014.

[50] G8 new alliance for food security and nutrition. Malawi. 2014. Annual progress report. Lilongwe: Ministry of Agriculture and Irrigation; 2014.

